# A Top-Down Approach to the Fabrication of Flame-Retardant Wood Aerogel with In Situ-Synthesized Borax and Zinc Borate

**DOI:** 10.3390/ma17112638

**Published:** 2024-05-30

**Authors:** Mingzeng Lin, Xiangkun Guo, Yinchao Xu, Xuejin Zhang, Donghao Hu

**Affiliations:** 1 School of Environmental and Natural Resources, Zhejiang University of Science and Technology, Hangzhou 310023, China; mingzenglin@126.com (M.L.); gxk729@126.com (X.G.); xuejinzhang@126.com (X.Z.); 2 School of Chemistry and Chemical Engineering, Shanghai Jiao Tong University, Shanghai 200030, China

**Keywords:** cellulose, wood aerogels, flame retardancy, composite materials

## Abstract

In this study, a top-down approach was employed for the fabrication of flame-retardant wood aerogels. The process involved the removal of lignin and the removal of hemicellulose utilizing NaOH concomitantly with the incorporation of ZnO and urea. Subsequently, an in situ reaction with boric acid was conducted to prepare flame-retardant wood aerogels. The morphology, chemical composition, thermal stability, and flame retardancy of the samples were studied. The results show that the NaOH treatment transformed the wood into a layered structure, and flame-retardant particles were uniformly distributed on the surface of the aerogel. The peak heat release rate (PHRR) and total heat release (THR) of the flame-retardant aerogel were significantly reduced compared with the control samples. Meanwhile, its vertical burning test (UL-94) rating reached the V-0 level, and the Limiting Oxygen Index (LOI) could exceed 90%. The flame-retardant wood aerogel exhibited excellent flame retardancy and self-extinguishing properties.

## 1. Introduction

Cellulosic raw materials originate from diverse sources and offer notable advantages, including biocompatibility, degradability, and renewability. The remarkable potential of cellulose has garnered significant attention from researchers worldwide, driving the exploration and development of numerous cellulose-based materials. Of particular interest among these cellulose-based materials are cellulose aerogels, distinguished by their unique three-dimensional porous network structure. Characterized by low density, high strength, substantial porosity, extensive surface area, low thermal conductivity, low thermal expansion, and sustainable attributes [1,2,3], cellulose aerogels find applications across diverse domains including medicine, energy, environmental remediation, and construction [3,4,5].

The fabrication process of biomass-based aerogels typically entails a series of intricate procedures encompassing chemical purification, high-temperature treatment, mechanical comminution, and subsequent refinement via specific machinery [6,7,8,9]. These processes, while indispensable for aerogel production, present formidable challenges due to demanding equipment requisites and elaborate extraction protocols [10]. Furthermore, the synthesis of cellulose aerogels necessitates dissolution dispersion, sol–gel transformations, and desiccation. Cellulose molecules, characterized by linear configurations intertwined within a robust hydrogen bonding network, exhibit insolubility in aqueous mediums, thus rendering the dissolution or dispersion of cellulose a foremost impediment in aerogel preparation. Presently, strategies such as the production of nanocellulose and the utilization of LiCl/DMAc solvent [11], as well as low-temperature agitation with NaOH/Urea/H_2_O [12], are commonly adopted to enhance the solubility and dispersibility of cellulose fibers. Subsequent gelation is achieved through physical or chemical means, followed by the removal of the liquid phase while preserving the three-dimensional network architecture, accomplished through freeze drying, supercritical drying, or ambient pressure drying techniques.

Traditionally, unmodified cellulose aerogels exhibit a loosely knit network structure with suboptimal mechanical properties. In addition to the aforementioned methodologies, a “top-down” approach is employed for direct delignification and hemicellulose removal from wood, thereby facilitating its transformation into aerogels. Wood, an economically viable and environmentally sustainable cellulose composite material, inherently possesses a preorganized molecular structure comprising cellulose fibers, hemicellulose, and lignin, intricately intertwined to form an open lumen and a natural hierarchical arrangement [13]. Leveraging the inherent hierarchical architecture of wood, the “top-down” preparation technique directly converts wood into cellulose aerogels, yielding high-performance anisotropic aerogels while mitigating energy consumption. Notably, natural wood, with its inherent porous and stratified configuration, has historically demonstrated exceptional thermal insulating properties. Nonetheless, the inherent mechanical frailty and flammability of cellulose aerogels curtail their applicability [14], necessitating concerted efforts to augment their mechanical robustness and flame retardancy for broader utilization.

So far, the flame retardancy of cellulose aerogels is generally enhanced by physically blending flame retardants or generating flame retardants in situ, such as adding aluminum hydroxide [15], silica [16], boric acid [17], etc. For decades, boric acid has been widely used as a flame retardant due to its easy commercial availability and environmentally friendly characteristics [18,19]. Boric acid can reduce the maximum degradation temperature and increase the char yield of wood biomass [20,21]. It is generally believed that boric acid mainly acts to inhibit heat and mass transfer during biomass pyrolysis [22]. In addition, some researchers have pointed out that boric acid can act as a catalyst for certain specific reactions, such as dehydration, isomerization, etc. [23]. Wang proposed that boric acid can catalyze not only the dehydration and deoxygenation reactions of wood at 100–300 °C but also catalyze the isomerization of newly formed intermediates, ultimately forming aromatic structures and char [22]. Zhang found that wood fibers undergo complexation and esterification reactions with boric acid, promoting the formation of char [24]. Furthermore, in order to improve the flame retardancy of CNF aerogels, researchers have also developed more complex in situ synthesis strategies, such as Nabipour [25], who grew ZIF-8 (Zeolitic Imidazolate Framework-8) in situ on the surface of fibers to prepare ZIF-8@cellulose composite aerogels. The addition of ZIF-8 enhanced the thermal stability, flame retardancy, and mechanical properties of cellulose aerogels. FAN [26] prepared CNF/AlOOH aerogels by hydrothermal method, where CNF can wrap AlOOH particles like a net, acting as a framework to prevent the aggregation of AlOOH. Ren [27] assembled melamine (MEL) and phytic acid (PA) in situ on cellulose CNF aerogels through supramolecular assembly.

Contemporary research has made significant strides in enhancing the flame retardancy of cellulose aerogels. Nonetheless, these endeavors encounter certain constraints, including reliance on costly materials and equipment, intricate and time-intensive procedures, as well as environmental ramifications. Hence, there exists a pressing imperative to devise a straightforward and viable approach for the fabrication of flame-retardant cellulose aerogels, thereby broadening their utility.

In this work, a novel nitrogen/boron/zinc cooperative in-situ flame retardant system was developed for wood aerogel. We have embraced a pragmatic yet efficacious “top-down” methodology employing Balsa wood as the primary substrate. The wood undergoes a “delignification” process utilizing a blend of NaClO_2_ and glacial acetic acid. Subsequently, the removal of hemicellulose is achieved through treatment with NaOH, concomitantly incorporating flame-retardant additives such as ZnO and urea. Ultimately, boric acid was then added to prepare in situ flame-retardant wood aerogel, forming an in-situ synergistic flame-retardant wood aerogel with outstanding flame-retardant performance. Notably, the NaOH treatment imbues the wood aerogel with a contoured and stratified architecture, endowing it with exceptional mechanical resilience and thermal insulating properties, thereby presenting promising avenues for utilization in flame-retardant and thermal insulation applications.

## 2. Materials and Methods

### 2.1. Materials

Balsa wood was purchased from Guangzhou Qigao Balsa Wood Trading Co., Ltd., Guangzhou, China; sodium chlorite, glacial acetic acid, sodium hydroxide, hydrochloric acid, and zinc oxide were purchased from Shanghai Lingfeng Chemical Reagent Co., Ltd., Shanghai, China; and urea and boric acid were purchased from Shanghai McKlin Biochemical Technology Co., Ltd., Shanghai, China.

### 2.2. Preparation of Flame-Retardant Wood Aerogel

The procedure for the preparation of flame-retardant wood aerogels is detailed as follows:

(1) Preparation of delignified wood (DW): Balsa wood is sectioned into varying dimensions, such as 15 mm × 15 mm × 15 mm, 10 mm × 100 mm × 5 mm. A 3% sodium chlorite solution is prepared, and its pH is adjusted to 4.5 using glacial acetic acid. Balsa wood specimens are immersed in the sodium chlorite solution and subjected to delignification at 95 °C within an oil bath. Delignification duration ranges from 6 to 24 h. Following the reaction, samples are thoroughly rinsed and subsequently frozen at −40 °C for 12 h. Freeze drying is then conducted using a vacuum freeze dryer for 48 h.
(2) Removal of hemicellulose: Separately, 6%, 8%, and 10% sodium hydroxide solutions are prepared. Zinc oxide particles are added to each solution in varying amounts, maintaining a mass ratio of NaOH to ZnO at 20:1. The mixture is heated and stirred in a water bath until complete dissolution. Subsequently, 4% urea is added to each solution to obtain a homogeneous mixture. Delignified wood obtained from step (1) is immersed in the respective solutions and subjected to a reaction at 90 °C for 5 h. Following the reaction, samples are frozen and subsequently freeze-dried.
(3) Reaction with Boric Acid Solution: A 10% boric acid solution is prepared. Samples treated with the sodium hydroxide–zinc oxide–urea solution are immersed in the boric acid solution and reacted at 80 °C in an oven for 24 h. After the reaction, samples are frozen and subjected to freeze drying, resulting in the preparation of flame-retardant wood aerogels.

**Figure 1:**
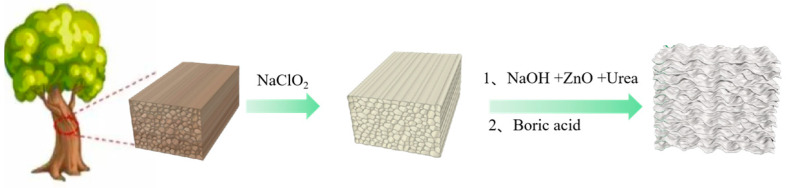
Preparation diagram of flame-retardant wood aerogel.

The preparation process of flame-retardant wood aerogel is shown in Figure 1.

For ease of writing, the samples are named as follows: NW (natural wood), DW (delignified wood), FRW6 (flame-retardant wood aerogel prepared from DW treated with 6% NaOH solution), FRW8 (flame-retardant wood aerogel prepared from DW treated with 8% NaOH solution), FRW10 (flame-retardant wood aerogel prepared from DW treated with 10% NaOH solution).

### 2.3. Characterization

Measurements of material dimensions are conducted utilizing a vernier caliper to ascertain length, width, and height, enabling the calculation of volume. Subsequently, employing a precision balance, the mass of the material is determined, from which the density (ρ) is computed using Equation ρ = m/V, where m represents the mass, and V represents the volume.

Scanning electron microscopy (SEM, SU 1510, Hitachi, Tokyo, Japan) equipped with energy-dispersive spectrometry (EDS, Element, EDAX, Pleasanton, CA, USA) was used for observation of the morphologies of the samples and elemental analysis. The samples were treated with Pt spraying before observation. The FTIR spectra were obtained by scanning the aerogel using an infrared spectrometer (BLUKER V70, Bruker Technology GMBH, Bremen, Germany). The samples were ground and then tested by KBr. The scanning times are 32 times/min, the scanning range is 800–3400 cm^−1^, and the resolution is 4 cm^−1^. X-ray Diffraction (XRD, D8 ADVANCE, Bruker Technology, Germany) was used to scan the aerogel to obtain XRD patterns using A Cu-Kα radiation source with a scanning range of 5–60° and a scanning speed of 4°/min. Thermogravimetric analysis was performed by using a thermogravimetric analyzer (TA Discovery Sdt650, Waters Technology, Shanghai, China) according to ASTM E2550 standard [28]. About 5 mg of the sample was heated at a heating rate of 10 °C/min from 30 °C to 700 °C under an N_2_ atmosphere. The top temperature of the sample was taken using an infrared thermal imager (323pro+, Fotric, Shanghai, China), The ambient temperature is 30 °C. The 10 mm thick sample was placed on an iron plate at 75 °C, and the temperature at the top of the sample was measured at different times. The Limiting Oxygen Index (LOI) test used an oxygen index meter (VOUCH 5801A, Suzhou Yangyi Vouch, Suzhou, China). The sample was kept at 25 ± 2 °C with 50 ± 5% humidity for 24 h, performed to assess the combustion behavior of samples according to ASTM D2863-2009 [29], and the dimension of the sample was 100 mm × 10 mm × 5 mm. UL-94 testing was conducted utilizing a horizontal–vertical burning test machine (VOUCH RS5402, Suzhou Yangyi Vouch, China) in accordance with ASTM D3801 standard [30], and the dimension of the sample was 100 mm × 10 mm × 5 mm. A microcombustion calorimeter (MCC-2, TA Instruments, New Castle, DE, USA) was employed in adherence to ASTM D7309-2007 standard [31]. Sample weighing between 5 mg was subjected to heating from 30 °C to 600 °C at a rate of 10 °C/min^−1^ under a pure nitrogen atmosphere. The flame-retardant aerogel was burned under a butane Bunsen burner for 30 s to observe the combustion state of the aerogel. Contact with the open flame part after burning creates carbon residue, which is used for carbon residue analysis. A thermogravimetric analyzer (TG 209 F1, Netzsch Scientific Instruments, Shanghai, China) is coupled with an infrared spectrometer (Nicolette IS 50, Thermo Fisher Scientific Inc., Waltham, MA, USA) to facilitate simultaneous TG-FTIR analysis. A sample weighing 10 mg, cut into fragments, is placed within the sample crucible of the TG instrument. Heating from ambient temperature to 800 °C at a rate of 10 °C/min under a pure nitrogen atmosphere ensues, with infrared spectrometry synchronously monitoring gases generated during sample pyrolysis. Acid-insoluble lignin content is quantified as per GB/T 2677.8-94 standard [32]. Conductivity titration methodology is employed to determine carboxyl group content.

## 3. Results and Discussion

### 3.1. Morphology and Chemical Structure of the Aerogels

**Figure 2:**
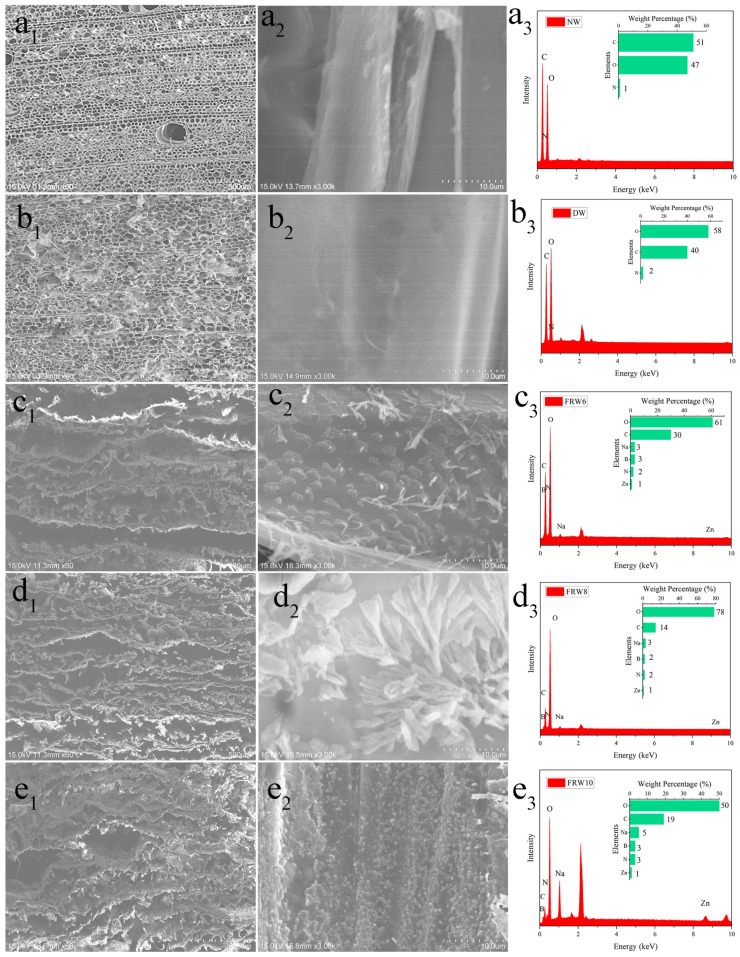
SEM image of NW (**a_1_**) cross-section, (**a_2_**) diameter section; DW (**b_1_**) cross-section, (**b_2_**) diameter section; FRW6 (**c_1_**) cross-section, (**c_2_**) diameter section; FRW8 (**d_1_**) cross-section, (**d_2_**) diameter section; FRW10 (**e_1_**) cross-section, (**e_2_**) diameter section, (**a_3_**–**e_3_**) is the EDS picture corresponding to (**a_2_**–**e_2_**).

The cutting method of the sample is shown in Figure S1. By observing different samples using a scanning electron microscope (SEM), the microstructure is shown in Figure 2. It can be observed that the cells of the Balsa wood are arranged in an oriented manner and exhibit a porous honeycomb structure in the cross-section, with close connections between the wood cells [33]. After treatment with NaClO_2_/acetic acid mixture for delignification, the cell walls become thinner and the luminal surface becomes smoother, but the oriented honeycomb structure of the wood remains unchanged. The density from the original ranges from 0.151 g/cm^3^ to 0.100 g/cm^3^, as shown in Table 1.

Subsequently, while removing hemicellulose with NaOH, flame-retardant ZnO and urea are introduced. After freeze-drying, the material reacts with the boric acid solution to in situ generate zinc borate and sodium borate. As shown in Figure 2(c_1_–e_1_), NaOH treatment transforms the original honeycomb structure into a layered structure, and the layered structure becomes looser with increasing NaOH concentration. From Figure 2(c_2_–e_2_), it is clear that a large number of flame-retardant particles are uniformly distributed on the surface of the wood aerogel, and the surface particles become denser with increasing NaOH concentration. This can be explained by the fact that NaOH treatment separates the cellulose nanofibers in the wood cell walls from each other, creating nanoscale voids. Zn^2+^ is adsorbed into the pores of the aerogel’s three-dimensional porous structure until absorption equilibrium is reached. Then, Zn^2+^ can interact with the hydroxyl groups on the cellulose via electrostatic interactions, resulting in the uniform distribution of zinc borate particles on the surface of the aerogel and within its pores [34]. EDS analysis of Figure 2(e_2_) shows that the main elements on the FRW10 are B, C, N, O, Na, and Zn, with respective contents of 3%, 19%, 3%, 50%, 5%, and 1%. Element B and Zn originate from zinc borate, C comes from fibers, N comes from urea, O comes from fibers and zinc borate, and Na comes from sodium borate. The flame retardants of flame-retardant aerogel can be detected in the EDS characterization.

**Figure 3:**
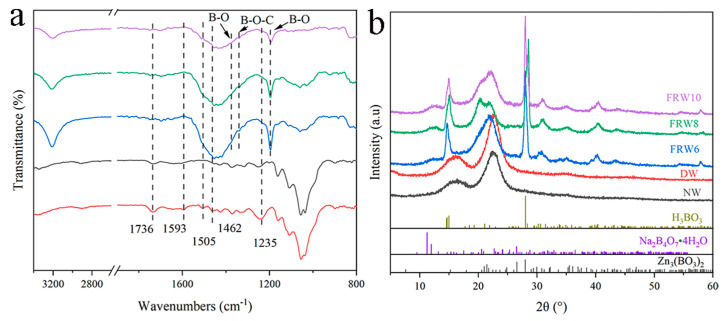
(**a**) FTIR spectra and (**b**) XRD spectra pattern of different samples.

According to the FT-IR spectrum, compared with NW, the spectrum of DW shows the disappearance of aromatic skeletal peaks at 1593, 1505, and 1462 cm^−1^, indicating the removal of lignin during the acidic NaClO_2_ treatment process (Figure 3). Additionally, the flame-retardant wood aerogel exhibits a significant decrease in the carbonyl stretching peak at 1736 cm^−1^ and the C-O stretching peak at 1235 cm^−1^ after treatment with NaOH/ZnO/Urea, reflecting the removal of hemicellulose [35]. Furthermore, the FT-IR spectrum strongly confirms the effective loading of the flame retardant. The absorption peak at 1375 cm^−1^ corresponds to the symmetric stretching vibration of the B-O bond, the absorption peak at 1340 cm^−1^ corresponds to the bridging stretching vibration of the B-O-C bond, and the absorption peak at 1195 cm^−1^ corresponds to the planar bending vibration of the B-O bond [23]. As shown in the XRD pattern, both NW and DW exhibit cellulose crystal structure of type I, which transforms into type II upon treatment with NaOH. Additionally, fitting peaks around 15° and 28° correspond to H_2_BO_3_, a characteristic peak caused by zinc borate Zn_3_(BO_3_)_2_ is detected around 33°, and a weak peak near 12° is mainly caused by Na_2_B_4_O_7_·4H_2_O. These results indicate that boric acid, borax, and zinc borate were successfully loaded onto the cellulose surface. In conclusion, the successful preparation of flame-retardant wood aerogel is demonstrated.

### 3.2. Mechanical Property Analysis

**Figure 4:**
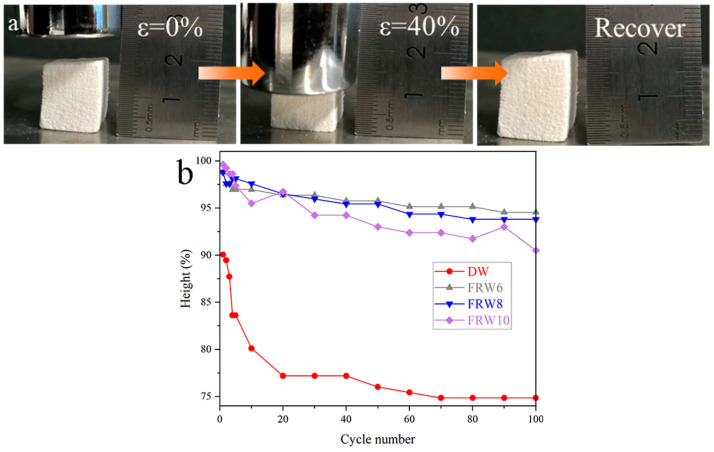
(**a**) Pictures of FRW10 compressing and releasing; (**b**) the constant strain is 40%, and there is a high retention rate in 100 compression/release cycles for different samples.

As shown in the figure, FRW10 releases pressure after 40% compression and returns to its original height. Under a constant strain of 40%, a series of 100 cyclic compression tests were performed on the DW, FRW6, FRW8, and FRW10. The cyclic number and the corresponding height retention rates of the aerogels are illustrated in Figure 4b. Following the completion of 100 cycles of 40% compression, the height retention rates, expressed as a percentage of the original height, were determined to be 74.85%, 90.81%, 90.39%, and 87.50% for the DW, FRW6, FRW8, and FRW10, respectively. These findings elucidate that the DW aerogel possesses a certain degree of compressibility and resilience. In contrast to the DW aerogel, the flame-retardant wood aerogels exhibit an improved resilience. The plastic deformation observed in the compressed DW aerogel stands in stark contrast to the heightened elasticity displayed by the flame-retardant wood aerogels. The DW aerogel retains its inherent honeycomb-like structure, which renders it susceptible to stress concentration under external loading conditions, thereby leading to structural failure and consequent plastic deformation. Conversely, the removal of hemicellulose through NaOH treatment imparts a more porous structure to the aerogels, while the in situ generation of flame retardants confers a supportive effect on the elasticity of the aerogels. Consequently, the flame-retardant aerogels exhibit enhanced resilience. However, it should be noted that the more aggressive structural damage induced by a higher concentration of NaOH exerts a discernible influence on the resilience of the aerogels.

### 3.3. Thermal Stability Analysis

**Figure 5:**
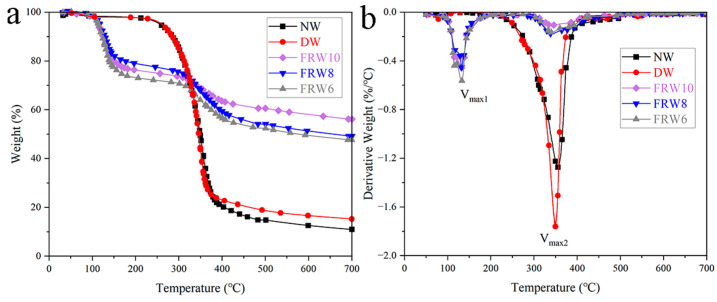
(**a**) TG; (**b**) DTG diagram of different samples. According to ASTM E2550 standard, samples were heated at a heating rate of 10 °C/min from 30 °C to 700 °C under an N_2_ atmosphere.

The TG and DTG curves of the samples in an N_2_ atmosphere are depicted in Figure 5. The thermal decomposition behavior of the raw wood commences at approximately 200 °C, with the principal pyrolysis range spanning from 230 to 480 °C. The maximum rate of weight loss (V_max_) is observed to be −1.28%/°C, corresponding to a temperature of 356.04 °C. At 700 °C, the residual carbon content reaches 11.02%. The thermal decomposition process of the raw wood can be delineated into three distinct stages: the initial stage (30–200 °C) primarily involves the elimination of low-molecular-weight volatile compounds, including water and organic substances, resulting in a minor mass loss of approximately 2%; the secondary stage (180–480 °C) predominantly entails the decomposition of cellulose, hemicellulose, and lignin, resulting in a weight loss of around 83%, signifying the principal degradation phase of the raw wood; the tertiary stage (480–700 °C) represents the carbonization phase, wherein lignin and nonvolatile, noncombustible constituents are converted into tar or char, leading to a weight loss of around 4% [23]. The flame-retardant wood aerogel exhibits two notable weight loss peaks, with the first peak occurring at around 120–130 °C, attributed to the dehydration of sodium borate, boric acid, and zinc borate crystals [17,36], as well as the thermal decomposition of urea, free water [37]. Compared with NW and DW, T_max_ decreases with the addition of flame retardants. This can be attributed to the catalytic action of boric acid, sodium borate, and zinc borate on the thermal degradation of fibers, which is a favorable phenomenon. Flame retardants catalyze the dehydration of cellulose and promote the formation of thermally stable carbon, resulting in the production of less volatile materials. Following the incorporation of flame retardants, a significant enhancement in the residual at 700 °C is observed for all samples (as presented in Table 2), with the FRW10 reaching a value of 56.16%. Additionally, the peak pyrolysis rate is substantially influenced by the introduction of flame retardants. For instance, during the primary degradation phase, the peak pyrolysis rates for the FRW6, FRW8, and FRW10 reduce from −1.28%/°C to −0.17%/°C, −0.18%/°C, and −0.11%/°C, respectively. This effect is potentially ascribed to the barrier properties of B_2_O_3_, the prompt formation of char facilitated by boron acid, and the dilution effect of ZnO and gas-phase crystallization water liberated by zinc borate. These flame-retardant mechanisms serve to safeguard the structural integrity of cellulose and decelerate its decomposition rate [38].

### 3.4. Thermal Insulation Analysis

**Figure 6:**
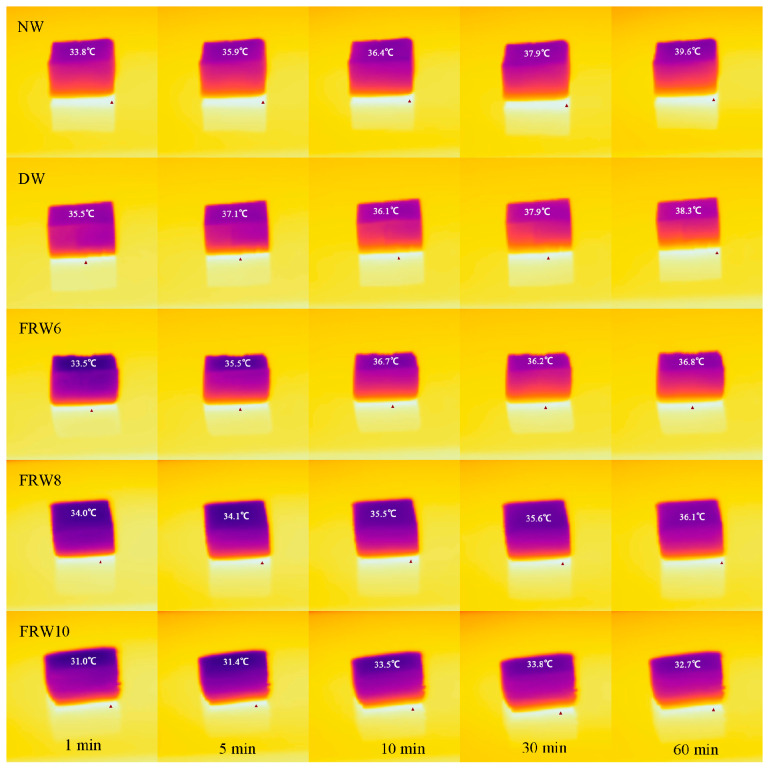
Thermal images of different samples. The ambient temperature is 30 °C. The 10 mm thick sample was placed on an iron plate at 75 °C.

Thermal insulation tests were performed on NW, DW, and FRW6, FRW8, and FRW10. A 10 mm thick aerogel sample was placed on a 75 °C iron plate, and a thermal imaging camera was employed to measure the surface temperature of the sample at a distance of 10 cm from the iron plate at different time intervals. Figure 6 shows the thermal image. Evidently, the FRW10 exhibits the most pronounced thermal insulation effect. After 60 min of heating, the top surface temperature of the aerogel reaches a mere 33.8 °C, accompanied by a temperature difference (Δ) of 41.2 °C (Δ = 75 °C heating temperature minus the top surface temperature of the aerogel). In contrast, the NW has a top surface temperature of 39.6 °C. This significant enhancement in thermal insulation performance can be attributed to the incorporation of NaOH, which induces a looser interlayer structure within the aerogel matrix. Furthermore, an intensified structural transition is observed as the NaOH concentration increases. This altered structure impedes the upward heat transfer from the underlying layers, consequently endowing the FRW10 with superior thermal insulation properties.

### 3.5. Combustion Analysis

**Figure 7:**
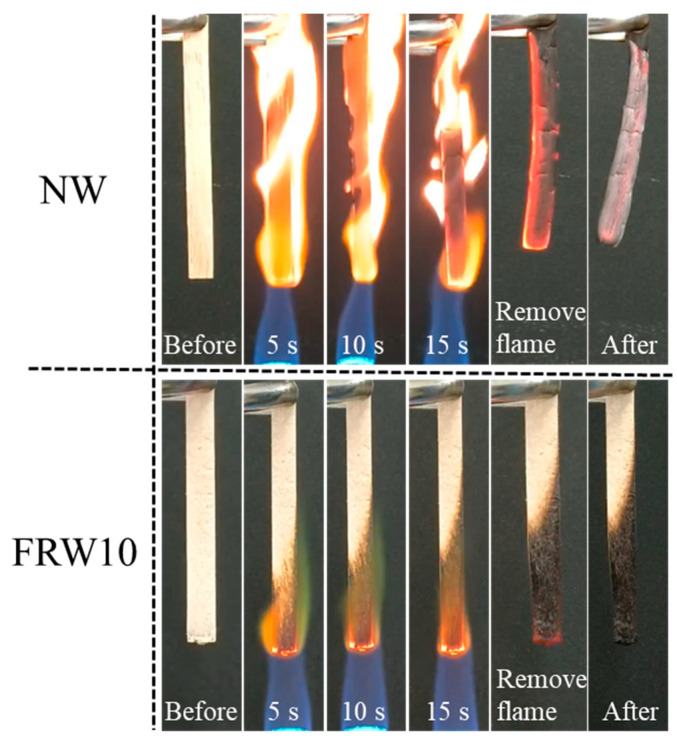
Burning diagram of NW and FRW10.

The flame retardancy of the aerogels was investigated using the direct flame ignition method with n-butane, as depicted in Figure 7. A comparative analysis of the combustion behavior between the NW and the FRW10 was conducted. The NW exhibited rapid ignition upon flame application and complete combustion within 15 s, leaving behind only a marginal amount of residual charred debris. It reveals the absence of any inherent flame-retardant properties of the NW. In contrast, the FRW10 swiftly formed a char layer on its surface following flame exposure. After a duration of 15 s, flame propagation was effectively curtailed, indicative of the aerogel’s self-extinguishing capability. Upon removal of the ignition source, the FRW10 promptly ceased to burn, underscoring its superior self-extinguishing characteristics. This behavior can be attributed to the loss of crystalline water from sodium borate and zinc borate during combustion, resulting in the dilution of ambient oxygen concentration and reduction in environmental temperature. Furthermore, the molten sodium borate and zinc borate function as heat absorbers during combustion, concurrently forming a protective layer on the surface of the aerogel. This protective layer effectively isolates oxygen and heat. Additionally, the presence of urea in the flame-retardant wood aerogel leads to the release of NH_3_ during heating, thereby diluting the concentration of oxygen around the aerogel. Consequently, the combustion conditions necessary for burning are not met, effectively suppressing the combustion process [39].

### 3.6. Flame-Retardant Performance Analysis

**Figure 8:**
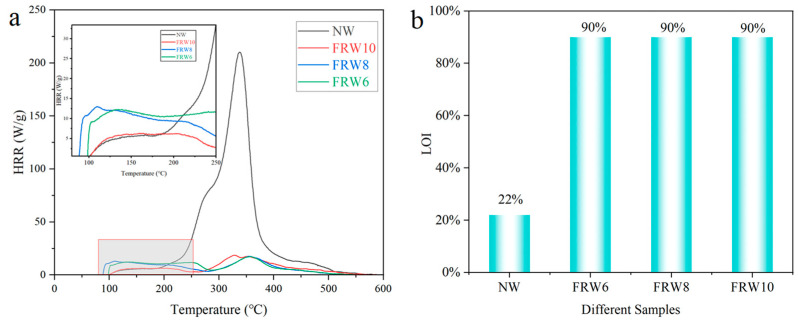
(**a**) HRR; (**b**) LOI diagram of different samples. According to ASTM D7309-2007 standard, the MCC test heats the sample from 30 °C to 600 °C at a rate of 10 °C/min^−1^ under a pure nitrogen atmosphere. According to ASTM D2863-2009, LOI test was carried out after the samples were kept at 25 °C ± 2 °C with 50% ± 5% humidity for 24 h.

The temperature-dependent heat release rates (HRR) of the NW and different flame-retardant composite aerogels were investigated using a microscale combustion calorimeter, as illustrated in Figure 8a. The measured data, including total heat release (THR), peak heat release rate (PHRR), and corresponding temperature (T_max_), are tabulated in Table 3. It was observed that the HRR of the NW reached 210.2 W/g at a temperature of 338.24 °C, implying the rapid liberation of substantial thermal energy. In the presence of flame retardants, a significant reduction in the PHRR values of the aerogels was discerned, with the FRW6, FRW8, and FRW10 exhibiting values of 17.3 W/g, 17.5 W/g, and 18.4 W/g, respectively. Concurrently, the THR values decreased from 18.7 kJ/g to 3.8 kJ/g, 3.6 kJ/g, and 3.4 kJ/g for FRW6, FRW8, and FRW10, respectively, corresponding to reductions of 79.7%, 80.7%, and 81.8%. Notably, within the low-temperature domain (<250 °C), the PHRR and THR of the FRW10 were approximately halved in comparison to the FRW6, FRW8. These findings collectively underscore the efficacy of sodium borate and zinc borate particles in mitigating cellulose combustion and suppressing the release of internal pyrolysis byproducts. The discerned heat release rate trends further corroborate the flame-retardant attributes inherent in the engineered wood aerogels. The incorporation of sodium borate and zinc borate significantly enhances the thermal stability and flame-retardant characteristics of wood aerogels.

The ignition properties of the wood aerogels were ascertained through Limiting Oxygen Index (LOI) and UL-94 assessments (Table 4). As illustrated in Figure 8b, the LOI value of the NW amounted to 22%, indicative of its susceptibility to ambient air ignition. In contrast, the LOI values of the FRW10 aerogels exceeded 90%, signifying an impressive enhancement of 309% relative to the NW and affirming a substantial improvement in flame retardancy. In the UL-94 test, the NW sample persisted in vigorous combustion even after the flame was extinguished, culminating in complete consumption within a time span of 35 s. Conversely, the flame persistence duration for the FRW6, FRW8, and FRW10 was a mere 0.8 s, with the consequent second flame and glowing durations amounting to 0.7 s and 0.1 s, respectively. Notably, all three flame-retardant aerogels gradually self-extinguished following flame removal, satisfying the V-0 classification criteria.

### 3.7. Flame-Retardant Mechanism Analysis

**Figure 9:**
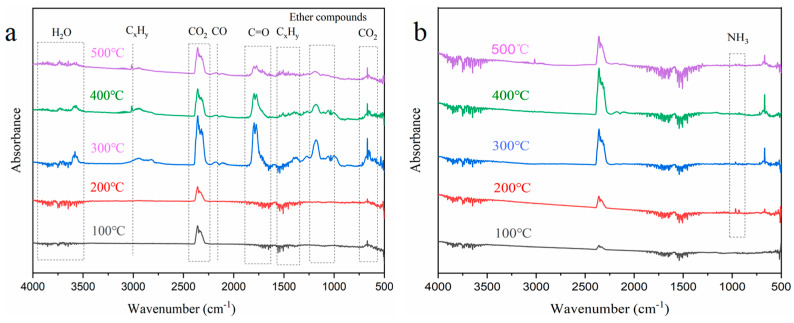
FTIR spectra of the released gaseous substances at different temperatures during the TG-FTIR test of (**a**) NW; (**b**) FRW10.

The thermal decomposition of the flame-retardant aerogel was analyzed using thermogravimetric analysis coupled with infrared spectroscopy (TG-IR). The results, as shown in Figure 9, reveal distinct peaks in the spectra of the NW precursor, indicating the release of various gaseous components. Specifically, peaks corresponding to H_2_O, aliphatic chains or hydrocarbon chains, CO_2_, CO, carbonyl compounds, ethers, and CO_2_ were observed in the wavenumber ranges of 3750–3500 cm^−1^, 3020–2790 cm^−1^, 2400–2260 cm^−1^, 2150 cm^−1^, 1870–1650 cm^−1^, 1232–1030 cm^−1^, and 750–600 cm^−1^, respectively [40,41]. The TG-IR analysis of the FRW10 indicated the release of these gaseous species during the experimental procedure, albeit with notable variations in release time and quantity. Additionally, a characteristic peak at 700–1100 cm^−1^, attributed to NH_3_, was observed exclusively in the FRW10 [42]. Upon heating, the volatile components from the NW were gradually released, exhibiting peaks for hydrocarbons, ethers, carbonyls, and CO. Notably, the peak release of these gases occurred around 300 °C. The general trend of gas evolution from the FRW10 was similar to that of the control sample, albeit with distinct variations in peak intensities. Specifically, the intensities of combustible gases, such as ethers, carbonyls, and CO, were significantly reduced, while the peak intensity of water vapor was notably enhanced. The peak release of gases was observed around 400 °C.

**Figure 10:**
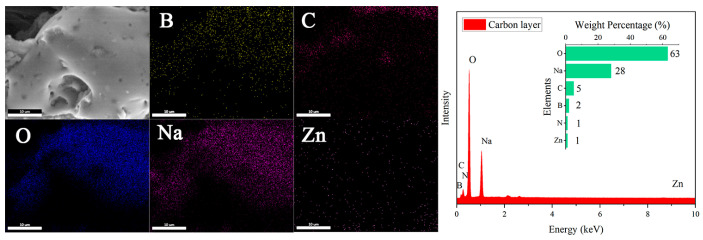
SEM and EDS diagram of FRW10 after burning.

SEM observation of the residual char obtained after the combustion of the FRW10 revealed minimal changes in the overall structure of the aerogel, while the flame-retardant particles on the surface disappeared (Figure 10). Instead, a continuous, dense, and expanded carbon layer was formed, and EDS showed that Na content increased significantly. This phenomenon can be attributed to the release of NH_3_ gas from urea upon heating, which, in conjunction with the molten sodium borate forming a protective layer, facilitated the expansion of the carbon layer. Furthermore, zinc borate decomposed into ZnO and B_2_O_3_, with B_2_O_3_ participating in the formation of a cross-linked network within the char, leading to increased density and effective isolation of gas exchange between the interior and exterior of the aerogel. These mechanisms contributed to improved flame retardancy and the self-extinguishing behavior of the flame-retardant aerogels.

**Figure 11:**
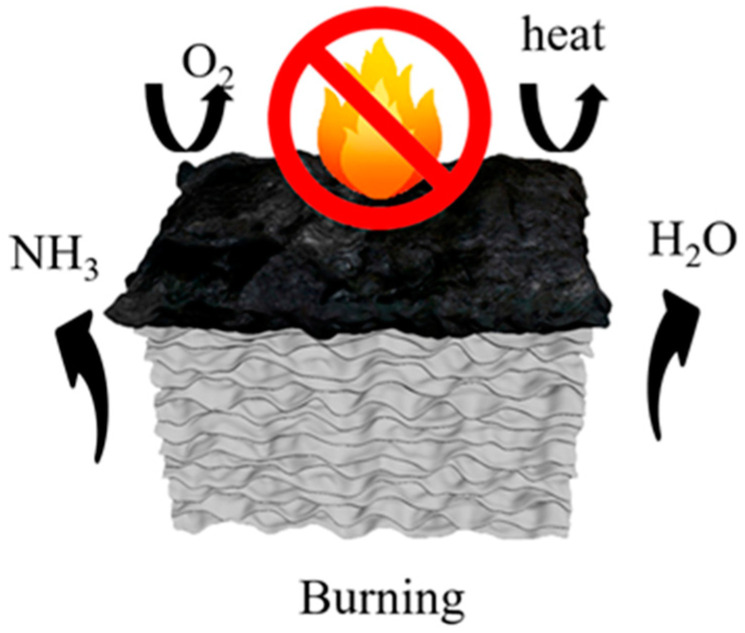
Aerogel flame-retardant mechanism diagram.

The flame-retardant mechanism is depicted in Figure 11. During the heating process, borax and zinc borate undergo dehydration, releasing water vapor that evaporates into the surroundings, thereby diluting flammable gases and absorbing heat. Notably, the combustion process does not generate any toxic, flammable, or corrosive gases. At approximately 160 °C, urea undergoes thermal decomposition, releasing NH_3_, which further dilutes the oxygen concentration in the surroundings. Subsequently, boron acid and zinc borate promote surface fiber carbonization, while molten borax forms a glassy protective layer on the aerogel surface. The resulting metal oxides, namely ZnO and B_2_O_3_, form a continuous and dense protective layer that retards heat propagation, suppresses the release of volatile combustible gases, and provides internal fiber protection [43]. Additionally, the inherent layered structure of the aerogel contributes to its heat barrier properties. As a result, the combustion process becomes hindered, leading to the self-extinguishing behavior of the aerogel.

## 4. Conclusions

A hierarchical composite flame-retardant aerogel with a layered structure was synthesized using a top-down approach by employing Balsa wood as the substrate, using sodium hydroxide to remove lignin and hemicellulose while adding zinc oxide and urea. Subsequently, an in situ reaction with boric acid is carried out to generate sodium borate flame retardant, preparing flame-retardant wood aerogel and utilizing the dual functions of NaOH. The microstructure and thermal behavior of the aerogel were comprehensively investigated. The characterization results demonstrated the uniform dispersion of flame-retardant additives throughout the porous network and on the surface of the aerogel. Notably, the structure of the aerogel remained essentially unchanged, even after undergoing combustion. Remarkably, the flame-retardant aerogel exhibited remarkable self-extinguishing capabilities upon flame removal, attesting to its superior flame retardancy. Mechanistically, during the combustion process, the presence of borax and zinc borate imparted a diluting effect on the surrounding oxygen due to the release of crystal water. Additionally, the melting of borate salts resulted in the formation of a protective layer, effectively retarding the decomposition of cellulose and concurrently suppressing the release of combustible gases during combustion. The peak heat release rate (PHRR) and total heat release (THR) of the flame-retardant aerogel were significantly reduced compared with the control samples. Meanwhile, its vertical burning test (UL-94) rating reached the V-0 level, and the Limiting Oxygen Index (LOI) could exceed 90%. The flame-retardant wood aerogel exhibited excellent flame retardancy and self-extinguishing properties.

## Figures and Tables

**Table 1:** Density of different samples.

Sample	NW	DW	FRW6	FRW8	FRW10
Density (g/cm^3^)	0.151	0.100	0.113	0.122	0.124

**Table 2:** TGA data of Balsa wood and different flame-retardant aerogels in N_2_.

Sample	NW	DW	FRW6	FRW8	FRW10
Residue (%)	11.02	15.22	47.69	49.27	56.16
T_10%_ (°C)	285.91	285.11	123.11	128.49	125.11
T_max1_ (°C)	-	-	132.79	131.92	132.18
V_max1_ (%/°C)	-	-	−0.56	−0.45	−0.45
T_max2_ (°C)	356.04	349.76	341.43	341.69	341.98
V_max2_ (%/°C)	−1.28	−1.76	−0.17	−0.18	−0.11

T_10%_ indicates the temperature at which the mass loss is 10 wt%; V_max_ and T_max_ indicate the speed and corresponding temperature at the maximum degradation rate, respectively.

**Table 3:** The MCC test results of the different aerogels in N_2_.

Sample	NW	FRW6	FRW8	FRW10
THR (KJ/g)	18.7	3.8	3.6	3.4
PHRR (W/g)	210.2	17.3	17.5	18.4
T_max_ (°C)	338.2	356.1	354.4	328

THR: total heat release, PHRR: peak heat release rate, T_max_: PHRR corresponding temperature.

**Table 4:** The UL94 test results of the different aerogels.

Sample	NW	FRW6	FRW8	FRW10
T1 (s)	35.0	0.9	0.9	0.8
T2 (s)	-	0.8	0.7	0.7
T3 (s)	-	0.1	0.1	0.1
Tf(T1 + T2) (s)	178.0	8.7	8.6	7.9
(T2 + T3) (s)	-	0.9	0.9	0.9
Whether residual flame or burn spreads to the fixture	YES	NO	NO	NO
Whether burning particles or droplets ignite cotton pads	NO	NO	NO	NO
UL-94	-	V-0	V-0	V-0

T1: Residual flame time after first ignition. T2: Residual flame time after second ignition. T3: Incandescent time after the first ignition. Tf(T1 + T2): Total residual flame time of 5 samples. (T2 + T3): Application of a second flame, residual flame time plus residual burning time.

## Data Availability

Data are contained within the article.

## Supplementary Materials

The following supporting information can be downloaded at https://www.mdpi.com/article/10.3390/ma17112638/s1, Figure S1. The cutting direction of the sample was observed before electron microscopy.

## References

[1] Gupta P., Singh B., Agrawal A.K. (2018). Low Density and High Strength Nanofibrillated Cellulose Aerogel for Thermal Insulation Application. Mater. Des..

[2] Zhang L., Liao Y., Wang Y. (2020). Cellulose II Aerogel-Based Triboelectric Nanogenerator. Adv. Funct. Mater..

[3] Ahankari S., Paliwal P., Subhedar A. (2021). Recent Developments in Nanocellulose-Based Aerogels in Thermal Applications: A Review. ACS Nano.

[4] Kamel R., El-Wakil N.A., Dufresne A. (2020). Nanocellulose: From an Agricultural Waste to a Valuable Pharmaceutical Ingredient. Int. J. Biol. Macromol..

[5] Xu T., Du H., Liu H. (2021). Advanced Nanocellulose-Based Composites for Flexible Functional Energy Storage Devices. Adv. Mater..

[6] Dhali K., Ghasemlou M., Daver F. (2021). A Review of Nanocellulose as a New Material towards Environmental Sustainability. Sci. Total Environ..

[7] Jiang F., Li T., Li Y. (2017). Wood-Based Nanotechnologies toward Sustainability. Adv. Mater..

[8] Lavoine N., Bergström L. (2017). Nanocellulose-Based Foams and Aerogels: Processing, Properties, and Applications. J. Mater. Chem. A.

[9] Chen W., Li Q., Wang Y. (2014). Comparative Study of Aerogels Obtained from Differently Prepared Nanocellulose Fibers. ChemSusChem.

[10] Sun H., Bi H., Lin X. (2020). Lightweight, Anisotropic, Compressible, and Thermally-Insulating Wood Aerogels with Aligned Cellulose Fibers. Polymers.

[11] Yang L., Meng J.G., Xue T. (2022). Research of Dissolution Process of Cotton Fiber in LiCl/DMAc Solvent. J. Text. Sci. Eng..

[12] Gao M., Cheng C.Z., Zhang D. (2021). Dissolution and Spinning Research progress of Cellulose in Alkali/Urea Compound Solvents at Low Temperature. Polym. Bull..

[13] Berglund L.A., Burgert I. (2018). Bioinspired Wood Nanotechnology for Functional Materials. Adv. Mater..

[14] Niu F., Wu N., Yu J. (2020). Gelation, Flame Retardancy, and Physical Properties of Phosphorylated Microcrystalline Cellulose Aerogels. Carbohydr. Polym..

[15] He C., Huang J., Li S. (2018). Mechanically Resistant and Sustainable Cellulose-Based Composite Aerogels with Excellent Flame Retardant, Sound-Absorption, and Superantiwetting Ability for Advanced Engineering Materials. ACS Sustain. Chem. Eng..

[16] Peng Q., Lu Y., Li Z. (2022). Biomimetic, Hierarchical-Ordered Cellulose Nanoclaw Hybrid Aerogel with High Strength and Thermal Insulation. Carbohydr. Polym..

[17] Yue X., Deng W., Zhou Z. (2023). Reinforced and Flame Retarded Cellulose Nanofibril/Sodium Alginate Compound Aerogel Fabricated via Boric Acid/Ca^2+^ Double Cross-Linking. J. Polym. Environ..

[18] Wang L., Sánchez-Soto M., Fan J. (2019). Boron/Nitrogen Flame Retardant Additives Cross-linked Cellulose Nanofibril/Montmorillonite Aerogels toward Super-low Flammability and Improved Mechanical Properties. Polym. Adv. Technol..

[19] Liu Q., Chai Y., Ni L. (2020). Flame Retardant Properties and Thermal Decomposition Kinetics of Wood Treated with Boric Acid Modified Silica Sol. Materials.

[20] Di Blasi C., Branca C., Galgano A. (2007). Flame Retarding of Wood by Impregnation with Boric Acid—Pyrolysis Products and Char Oxidation Rates. Polym. Degrad. Stab..

[21] Salman S., Pétrissans A., Thévenon M.F. (2014). Development of New Wood Treatments Combining Boron Impregnation and Thermo Modification: Effect of Additives on Boron Leachability. Eur. J. Wood Prod..

[22] Wang Q.L., Wang Q.L. (2004). Chemical Mechanism of Fire Retardance of Boric Acid on Wood. Wood Sci. Technol..

[23] Hou X., Li Z., Zhang Z. (2021). Selectively Producing Acetic Acid via Boric Acid-Catalyzed Fast Pyrolysis of Woody Biomass. Catalysts.

[24] Zhang J., Koubaa A., Xing D. (2020). Conversion of Lignocellulose into Biochar and Furfural through Boron Complexation and Esterification Reactions. Bioresour. Technol..

[25] Nabipour H., Nie S., Wang X. (2020). Highly Flame Retardant Zeolitic Imidazole Framework-8@cellulose Composite Aerogels as Absorption Materials for Organic Pollutants. Cellulose.

[26] Fan B., Chen S., Yao Q. (2017). Fabrication of Cellulose Nanofiber/AlOOH Aerogel for Flame Retardant and Thermal Insulation. Materials.

[27] Ren X., Song M., Jiang J. (2022). Fire-Retardant and Thermal-Insulating Cellulose Nanofibril Aerogel Modified by In Situ Supramolecular Assembly of Melamine and Phytic Acid. Adv. Eng. Mater..

[28] (2017). Standard Test Method for Thermal Stabilityby Thermogravimetry.

[29] (2009). Standard Test Method for Measuring the Minimum Oxygen Concentration to Support Candle-like Combustion of Plastics (Oxygen Index).

[30] (2020). Standard Test Method for Measuring the Comparative Burning Characteristics of Solid Plastics in a Vertical Position.

[31] (2007). Standard Test Method for Determining Flammability Characteristics of Plastics and Other Solid Materials Using Microscale Combustion Calorimetry.

[32] (1994). Fibrous Raw Material. Determination of Acid-Insoluble Lignin.

[33] Zhu G., Zhang C., Li K. (2023). A Multifunctional Zeolitic Imidazolate Framework-8@wood Aerogel Composite Intergrating Superior Performance of Dye Adsorption Capacity and Flame-Retardant Property. J. Porous Mater..

[34] Qin Q., Guo R., Ren E. (2020). Waste Cotton Fabric/Zinc Borate Composite Aerogel with Excellent Flame Retardancy. ACS Sustain. Chem. Eng..

[35] Zhu Z., Fu S., Lucia L. (2019). A Fiber-Aligned Thermal-Managed Wood-Based Superhydrophobic Aerogel for Efficient Oil Recovery. ACS Sustain. Chem. Eng..

[36] Tanpichai S., Phoothong F., Boonmahitthisud A. (2022). Superabsorbent Cellulose-Based Hydrogels Cross-Liked with Borax. Sci. Rep..

[37] Younis A.A. (2020). Optimization of Mechanical, Thermal, and Ignition Properties of Polyester Fabric Using Urea and Phosphoric Acid. J. Ind. Text..

[38] Cheng X., Zhu S., Pan Y. (2020). Fire Retardancy and Thermal Behaviors of Cellulose Nanofiber/Zinc Borate Aerogel. Cellulose.

[39] Hao D., Wang R., Wang W.Q. (2021). Application and Progress of Boron-Based Flame Retardants in Flame Retardant Polymers. Polym. Mater. Sci. Eng..

[40] Bai X.Y., Che D.Y., Jiang W.Q. (2015). TG-FTIR analysis of cellulose pyrolysis. Renew. Energy Resour..

[41] Luo X., Li Z., Shen J., Liu L., Chen H., Hu Z., Krucinska I., Yao J. (2022). A Facile Strategy to Achieve Efficient Flame-Retardant Cotton Fabric with Durable and Restorable Fire Resistance. Chem. Eng. J..

[42] Wang J., Du C.W., Shen Y.Z. (2014). Measurement of Ammonia in Soil Headspace by Mid-infrared Photoacoustic Spectroscopy. Soils.

[43] Wang Z., E Y., Li J., Du T., Wang K., Yao X., Jiang J., Wang M., Yuan S. (2023). Sustainable Bacterial Cellulose-Based Composite Aerogels with Excellent Flame Retardant and Heat Insulation. Cellulose.

