# Supplementary material for: A Top-Down Approach to the Fabrication of Flame-Retardant Wood Aerogel with In Situ-Synthesized Borax and Zinc Borate
Source: Materials (Basel). 2024 May 30;17(11):2638. doi: 10.3390/ma17112638 (PMC11173988; doi:10.3390/ma17112638)
Supplement: Supplementary file 1 [file materials-17-02638-s001.zip › materials-2998987-supplementary.pdf]

NW was cut untreated, DW was cut after delignification treatment, and FRW10 was cut after boric acid was added.

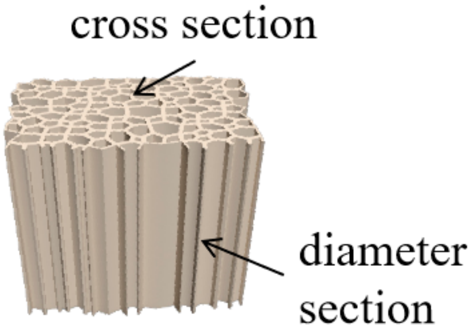

Figure S1. The cutting direction of the sample was observed before electron microscopy.
